# Will a Culturally Adapted Training Material for Caregivers Improve Dementia Care in Ghana?

**DOI:** 10.1002/alz.091715

**Published:** 2025-01-09

**Authors:** Sally Yalley, Kwaku Sarfo Manu, Roman Romero‐Ortuno

**Affiliations:** ^1^ Alzheimers and Related Association of Ghana, Accra Ghana; ^2^ Global Brain Health Institute, Trinity College, Dublin Ireland; ^3^ University of Ghana Medical Center, Accra Ghana; ^4^ Alzheimer’s and Related Disorders Association of Ghana, Accra Ghana; ^5^ Imperial College, London United Kingdom; ^6^ Mercer’s Institute for Successful Ageing, St James’s Hospital, Dublin Ireland; ^7^ Global Brain Health Institute (GBHI), Trinity College Dublin, Dublin Ireland

## Abstract

The World Health Organization (WHO) emphasizes the need to provide support to caregivers of people living with dementia, as one of seven action areas. This is especially crucial in Ghana, where, similar to other Lower Middle‐Income Countries (LMICs), the majority of people with dementia reside in the community and are cared for by informal caregivers, often family members. These caregivers usually do not have any training in dementia care and may have limited understanding of the disease and its potential symptoms. This increases the physical and psychological burden on caregivers, highlighting the urgent need for education and training of informal caregivers in Ghana (Ayisi‐Boateng et al. 2022).

The Alzheimer’s and Related Disorders Association of Ghana operates a caregiver support platform that has expanded since its inception. This aims to provide individualized support and education for caregivers, primarily utilizing freely available or open‐source educational resources such as the WHO iSupport manual, as well as educational materials from organizations like the Alzheimer’s Society UK, Alzheimer’s Association of the USA, and Alzheimer’s Disease International.

While the currently available materials offer a much‐needed foundational understanding, addressing translational and cultural differences is essential. Ghanaian cultural practices, marked by its heterogeneity and distinctions from Western culture, presents unique challenges in dementia care. Moreover, considering varying levels of literacy, particularly among older adults, the adaptation of resources to include tailored video content could be beneficial for a more inclusive and effective approach.

The WHO’s iSupport manual has been successfully translated and adapted into several languages and cultural settings around the world. Replicating this adaptation in the Ghanaian cultural context would be a momentous step in developing bespoke and culturally relevant dementia care resources in Africa.

